# Deep Learning Reconstruction Specialized for Inner Ear: Improving Image Quality and Anatomical Structure Visualization as Compared with Conventional Hybrid-Type Iterative Reconstruction on High-Definition CT

**DOI:** 10.3390/diagnostics16121756

**Published:** 2026-06-06

**Authors:** Masahiko Nomura, Hirona Kimata, Yuya Ito, Kenji Fujii, Naruomi Akino, Takahiro Ueda, Takeshi Yoshikawa, Daisuke Takenaka, Yoshiyuki Ozawa, Yoshiharu Ohno

**Affiliations:** 1 Department of Diagnostic Radiology, School of Medicine, Fujita Health University, 1-98 Dengakugakubo, Kutsukake-cho, Toyoake 470-1192, Aichi, Japan; nomura@fujita-hu.ac.jp (M.N.); t-ueda@fujita-hu.ac.jp (T.U.); takeshi.yoshikawa@fujita-hu.ac.jp (T.Y.); daisuke.takenaka.fr@fujita-hu.ac.jp (D.T.); yoshiyuki.ozawa@fujita-hu.ac.jp (Y.O.); 2 Joint Research Laboratory of Advanced Medical Imaging and Artificial Intelligence, School of Medicine, Fujita Health University, Toyoake 470-1192, Aichi, Japan; 3 Canon Medical Systems Corporation, Otawara 324-8550, Tochigi, Japan; hirona1.kimata@medical.canon (H.K.); yuya1.ito@medical.canon (Y.I.); kenji3.fujii@medical.canon (K.F.); naruomi.akino@medical.canon (N.A.); 4 Department of Radiology, Fujita Health University Bantane Hospital, Nagoya 454-8509, Aichi, Japan; 5 Department of Radiology, Fujita Health University Okazaki Medical Center, Okazaki 444-0827, Aichi, Japan

**Keywords:** ear, computed tomography, x-ray, artificial intelligence, reconstruction

## Abstract

**Background/Objectives:** To directly compare the capabilities of hybrid-type iterative reconstruction (IR) with the newly developed deep learning reconstruction (DLR) for the inner ear on high-definition CT (HDCT) obtained using the super-high-resolution (SHR) mode for external, middle and inner ear evaluations and diagnosis in patients with and without otologic diseases. **Methods:** Included in this study were 140 patients who had undergone HDCT, consisting of 32 otologic disease patients and 108 non-otologic disease patients, and 280 inner and middle ears and temporal bones were evaluated on a per ear analysis. Signal-to-noise ratios (SNRs) of the temporal bone surrounding the aural vestibule of the ear and in the vestibule as well as the cerebellar hemisphere, overall image and detailed evaluation of the visibility of anatomical landmarks in the middle and inner ear and temporal bone obtained with the two methods were assessed and statistically compared using the paired *t*-test or Wilcoxon’s signed-rank test. Then, receiver operating characteristic (ROC) analysis was performed to compare diagnostic performance between two reconstruction methods. **Results:** Each SNR of DLR was significantly higher than that of hybrid-type IR (*p* < 0.05). Overall image quality and detailed visualization of each anatomical structure obtained with DLR were significantly better than those obtained with hybrid-type IR (*p* < 0.05). The area under the curve of DLR had no significant difference with hybrid-type IR (*p* = 0.18). **Conclusions:** DLR has superior potential to hybrid-type IR for better image quality and visualization of anatomical landmarks in middle and inner ears and temporal bones on HDCT, although diagnostic performance was not affected in clinical practice.

## 1. Introduction

Many anatomic structures of the middle and inner ear are not optimally depicted as 3D images on computed tomography (CT) using image reconstruction in the standard axial and coronal planes as well as volume rendering (VR). Recent advances in multidetector CT (MDCT), including the development of ultra-high-resolution CT using energy-integrated detectors also known as high-definition CT (HDCT) and photon counting detection (i.e., photon counting detector CT or PCDCT), allow for the acquisition of isotropic voxels measuring 0.25 mm or less that can be obtained and reconstructed in any plane of a section [1,2,3,4,5,6]. This technique enables radiologists to visualize the anatomic structures of the middle and inner ear (the ossicular chain, stapedial footplate—oval window complex, round window, cochlea, vestibular aqueduct, and bones of the superior semicircular canal and facial nerve canal) in greater detail and may help increase the accuracy of CT for the diagnosis of diseases of the external, middle and inner ear.

Since the 2010s, state-of-the-art reconstruction methods, such as hybrid-type iterative reconstruction (IR), model-based IR (MBIR) and deep learning reconstruction (DLR), have been introduced and used for CT examinations for various clinical purposes involving not only the ear but also other organs and have also begun to be used for not only MDCT, but also HDCT or PCDCT [1,2,3,4,5,6,7,8,9,10,11,12,13,14,15,16]. Although MBIR allows for more reduction in noise and artifacts than filtered back projection (FBP) and hybrid-type IR do, the high computational requirements and long reconstruction times, as well as specific artifacts due to over-smoothing and texture changing of MBIR, have limited its widespread clinical application [17,18]. Therefore, a new reconstruction method to overcome MBIR limitations and provide higher image quality than FBP and hybrid-type IR has been required for routine CT examination. This situation has prompted Canon Medical Systems Corporation to develop specialized DLR for the inner ear, with testing beginning in 2023. However, DLR specialized for the inner ear on HDCT has not been compared with hybrid-type IR and DLR used for various ear diseases. We hypothesized that such specialized DLR can yield better image quality, diagnostic confidence level or abnormality detection for the ear than other reconstruction methods on HDCT, which has three different scan modes: normal resolution (NR: 0.5 mm × 80 rows/896 channels), high resolution (HR: 0.5 mm × 80 rows/1792 channels) and super high resolution (SHR: 0.25 mm × 160 rows/1792 channels). The purpose of this study was to directly compare the capabilities of hybrid-type IR with DLR on HDCT obtained using the SHR mode for external, middle and inner ear evaluations and diagnosis in patients with and without otologic diseases.

## 2. Materials and Methods

This retrospective study was approved by the Institutional Review Board of Fujita Health University Hospital, is compliant with the Health Insurance Portability and Accountability Act, and written informed consent was waived. This study was technically and financially supported by Canon Medical Systems Corporation. Four of the authors are employees of Canon Medical Systems Corporation (Y.I., H.K., K.F. and N.A.) who did not have control over any of the data used in this study. Two of the authors (M.N. and Y.Oh) received a research grant from Canon Medical Systems, and an author (Y.Oh) also received a research grant from the Smoking Research Foundation. All data were controlled and statistically analyzed by M.N., T.U., T.Y., D.T., Y.O. and Y.Oh.

### 2.1. Subjects

**Figure 1:**
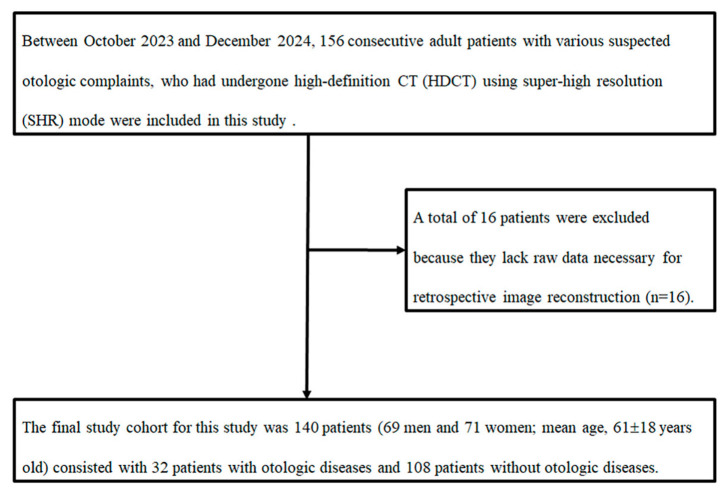
Patients’ selection flowchart for this study.

Included in this study were 312 inner and middle ears and temporal bones of 156 consecutive adult patients with suspected various otologic complaints, who had undergone HDCT using the SHR mode between October 2023 and December 2024. Sixteen out of 156 patients were excluded due to lacking raw data necessary for retrospective image reconstruction. The final study cohort for this study was 140 patients (69 men and 71 women; mean age, 61 ± 18 years old) and consisted of 32 patients with otologic diseases and 108 patients without otologic diseases. The flow chart for patient selection is shown in Figure 1. Patients’ characteristics were shown in Table 1.

### 2.2. CT Examinations

All patients underwent temporal CT using the helical scan mode as well as the SHR mode for a 160-detector row HDCT system (Aquilion Precision: Canon Medical Systems Corporation, Otawara, Tochigi, Japan). Acquisition parameters were: detector collimation, 0.25 mm × 160 row; tube potential, 120 kVp; tube current, automatic exposure control with noise index determined by image standard deviation (SD) as 6.5; pitch factor, 0.56; x-ray focus size, 0.4 × 0.5 mm; number of channels, 1792; rotation time, 1.0 sec/rot; slice thickness, 0.25 mm; slice increment, 0.125 mm; reconstruction matrix, 512 × 512 matrix. All CT data were reconstructed by means of hybrid-type IR (AIDR 3D: Canon Medical Systems) and DLR [Advanced intelligent Clear-IQ Engine for inner ear (AiCE inner ear)]: Canon Medical Systems).

### 2.3. Image Analysis

Both objective and subjective image quality were assessed for 108 patients who were diagnosed as having no evidence of otologic diseases in a blinded and randomized fashion on a picture archiving and communication system (RapideyeCore TFS01: Canon Medical Systems). To this end, image datasets were displayed in a random order using the standard bone window level of 4000 Hounsfield Units (HU) with a center of 300 HU. All technical and personal data were removed from the images.

### 2.4. Quantitative Image Analysis

For quantitative assessment, a board-certified radiologist (T.Y.) with 31 years of experience manually placed regions of interest (ROIs) over the temporal bone surrounding the bilateral vestibular region of the ear and in the vestibule as well as the cerebellar hemisphere of all patients. Image noise was also determined by placing circular ROIs on the same sides to measure the standard deviation of the CT value shown as background noise on temporal bone CT images obtained by means of HDCT. The signal-to-noise ratio of each anatomical lesion was then determined with the following formula:

(1) SNRtemporalbone,vestibulaorcerebellarhemisphere=Mean CT value measured in ROIImage noise

### 2.5. Qualitative Image Analysis

Qualitative image quality assessments were initially assessed separately by two other board-certified radiologists (Y.Oz and Y.Oh) with 22 and 32 years of experience, respectively, and later in a consensus reading session. Both radiologists rated overall image quality for each patient using the following 5-point visual scoring system: 1, poor; 2, fair; 3, moderate; 4, good; and 5, excellent. Artifacts were also rated with another 5-point visual scoring system: 1, excellent; 2, good; 3, moderate; 4, fair; and 5, poor.

Moreover, the accessibility of the middle ear, inner ear, and temporal bone structures was then rated as specified for temporal bone CT examinations in the European Guidelines on Quality Criteria for CT [19,20]. Visibility of anatomical landmarks in middle and inner ears and temporal bone was evaluated in detail using a 5-point visual scoring system: 1, poor; 2, fair; 3, moderate; 4, good; and 5, excellent. For the middle ear, the epitympanic recess, tegmen tympani, sinus tympani, facial recess, facial canal, cochleariform process, incudomalleal joint, incudostapedial joint, long process of the incus, crura of the stapes, and head and footplate of the stapes were evaluated. For the inner ear, fissula ante fenestram, modiolus, cochlear partition, round window, otic capsule homogeneity, semicircular canals, cochlear and vestibular aqueduct were assessed, as were the location of the jugular bulb, course of facial nerve, chorda tympani in mastoid bone, sigmoid sinus, and mastoid air cell aeration in the temporal bone.

In contrast to image quality evaluations, a total of 140 patients with 32 patients with otologic diseases and 108 patients without otologic diseases were evaluated for diagnostic confidence level on a per-patient basis by a 5-point scale as follows: 1, very unlikely; 2, unlikely; 3, may represent; 4, likely; and 5, most likely [21,22].

### 2.6. Statistical Analysis

The paired *t*-test was used to compare each quantitative image index on CT data sets obtained with hybrid-type IR and DLR, CT value and SNR at the temporal bone surrounding the vestibule of the ear, in the vestibule itself, and the cerebellar hemisphere.

Kappa statistics were used to assess inter-observer agreement for each qualitative index in all CT data sets. Agreements were considered slight for κ < 0.21, fair for κ = 0.21–0.40, moderate for κ = 0.41–0.60, substantial for κ = 0.61–0.80, and almost perfect for κ = 0.81–1.00 [23].

For a comparison of overall image quality and artifacts on CT data sets obtained with hybrid-type IR and DLR, Wilcoxon’s signed-rank test was employed. The same test was also used for a comparison of detailed evaluation of the visibility of anatomical landmarks and structures in the middle and inner ears and temporal bone obtained with hybrid-type IR and DLR for each CT data set.

To compare the diagnostic performance of each HDCT, inter-observer agreement for diagnostic confidence level on each reconstruction method was also assessed by kappa statistics. Then, receiver operating characteristic (ROC) analysis was performed to compare diagnostic performance between two reconstruction methods. Finally, sensitivity, specificity and accuracy were compared between two methods by McNemar’s test.

A priori power analysis was conducted to determine the required sample size. To detect a medium effect size (d = 0.50) with a statistical power of 0.80 at a significance level of (α = 0.05), the analysis indicated that a total of 26 participants were required for a two-tailed paired *t*-test. A power analysis was conducted to determine the required sample size for the ROC analysis. To achieve a statistical power of 0.80 with a two-sided significance level of (α = 0.05), and assuming an expected area under the curve (AUC) of 0.8, a minimum total sample of 26 participants (13 positive and 13 power negative cases) was required. For all statistical analyses using commercially available software (JMP 14: SAS Institute Japan, Co. Ltd., Tokyo, Japan; StatMate III: Atoms Co. Ltd., Tokyo, Japan) and two free software programs (EZR ver. 1.54: https://www.jichi.ac.jp/saitama-sct/SaitamaHP.files/statmedEN.html; and G*Power version 3.1.9.7, Franz Faul, Universität Kiel, Germany) [24,25], a *p*-value less than 0.05 was considered statistically significant.

## 3. Results

**Figure 2:**
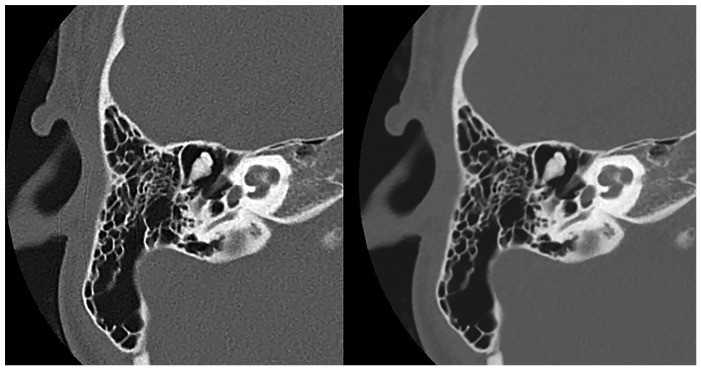
HDCT images at the inner ear level reconstructed by means of hybrid-type IR and DLR methods. (**Left**: hybrid IR, **Right**: DLR). Significant image noise and artifact reductions and improvements in SNR and overall image quality were confirmed by comparing HDCT images reconstructed with hybrid-type IR and with DLR, although the visibility of anatomical landmarks had little effect.

**Figure 3:**
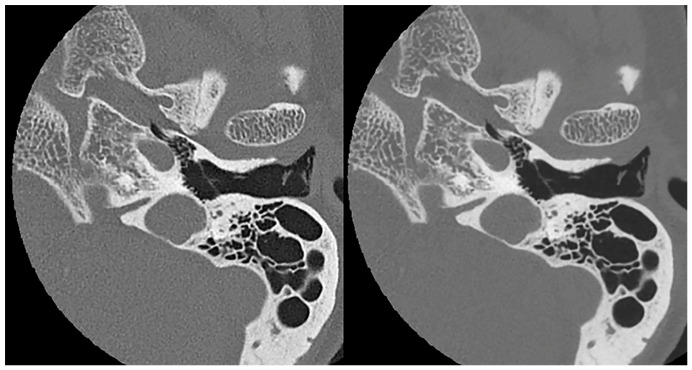
HDCT images at the middle ear level reconstructed by means of hybrid-type IR and DLR methods. (**Left**: hybrid IR, **Right**: DLR). Significant image noise and artifact reductions and improvements in SNR and overall image quality were confirmed by comparing HDCT images reconstructed with hybrid-type IR and with DLR, although the visibility of anatomical landmarks had little effect.

**Figure 4:**
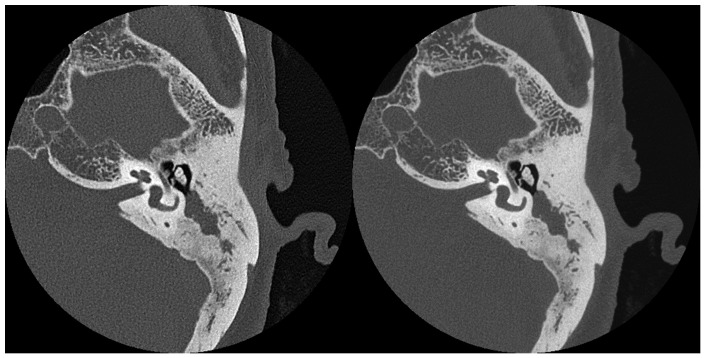
HDCT images in an otosclerosis patient at the inner ear level reconstructed by means of hybrid-type IR and DLR methods. (**Left**: hybrid IR, **Right**: DLR). Significant image noise and artifact reductions and improvements in SNR and overall image quality were confirmed by comparing HDCT images reconstructed with hybrid-type IR and with DLR. HDCT image reconstructed with DLR demonstrates incus synostosis and calcification of the stapedius tendon more clearly than that with hybrid-type IR. The diagnostic confidence level of the HDCT image reconstructed with DLR was 5, although that with hybrid-type IR was 4.

Findings for a representative case are shown in Figure 2, Figure 3 and Figure 4.

On comparison of quantitative indexes obtained with hybrid-type IR and DLR for CT data sets, DLR yielded significantly higher CT values for the temporal bone (1951.5 ± 73.4 HU), vestibule (65.0 ± 2.5) and cerebellar hemisphere (35.5 ± 15.4) than hybrid-type IR (temporal bone: 1841.9 ± 112.6 HU, *p* < 0.0001, vestibule: 61.4 ± 3.8 HU, *p* < 0.0001, cerebellar hemisphere: 40.9 ± 13.5 HU, *p* = 0.04). Moreover, SNRs of DLR for temporal bone (37.9 ± 11.9), vestibule (1.3 ± 0.4) and cerebellar hemisphere (0.7 ± 0.4) were significantly higher than those of hybrid-type IR (temporal bone: 13.8 ± 2.1, *p* < 0.0001, vestibule: 0.5 ± 0.1, *p* < 0.0001, cerebellar hemisphere: 0.3 ± 0.1, *p* < 0.0001).

Inter-observer agreements for overall image quality and artifacts are shown in Table 2. Assessment of the overall image quality of each CT data set showed inter-observer agreements for CT data reconstructed by DLR and hybrid-type IR were ‘substantial’ (DLR: κ = 0.61, *p* < 0.0001; hybrid-type IR: κ = 0.63, *p* < 0.0001), while inter-observer agreements for artifacts were rated ‘almost perfect’ or ‘substantial’ for CT images obtained with DLR (κ = 1.00, *p* < 0.0001) and hybrid-type IR (κ = 0.63, *p* < 0.0001).

When comparing the overall image quality and artifacts between two reconstruction methods, overall image quality and artifacts of CT data reconstructed by means of DLR (overall image quality: median, 5; interquartile range [IQR], 5–5; artifact: median, 1; IQR, 1–1) were significantly better than those reconstructed with hybrid-type IR (overall image quality: median, 4; IQR, 4–5, *p* < 0.0001; artifacts: median, 1; IQR, 1–2, *p* < 0.0001).

Inter-observer agreements for detailed evaluation of the visibility of anatomical landmarks in the middle and inner ear and temporal bone are shown in Table 3. Inter-observer agreements for detailed evaluation of the visibility of anatomical landmarks in the middle and inner ear and temporal bone were assessed as ranging from significant to ‘substantial’ (0.62 ≤ κ ≤ 0.80, *p* < 0.0001).

On comparisons of detailed evaluation of the visibility of anatomical landmarks in middle and inner ear and temporal bone, CT data for middle ear (median: 5, interquartile range [IQR]: 5–5), inner ear (median: 5, IQR: 5–5) and temporal bone (median: 5, IQR: 5–5) reconstructed by means of DLR scored significantly higher than those reconstructed by means of hybrid-type IR (middle ear: median, 4, IQR: 4–5, *p* < 0.0001; inner ear: median, 4, IQR: 4–5, *p* < 0.0001; temporal bone: median, 4, IQR: 4–5, *p* < 0.0001).

When evaluated for inter-observer agreement of diagnostic confidence level for each reconstruction method, agreements of DLR and hybrid-type IR were determined as ‘substantial’ (DLR: κ = 0.73, *p* < 0.0001; hybrid-type IR: κ = 0.70, *p* < 0.0001).

**Figure 5:**
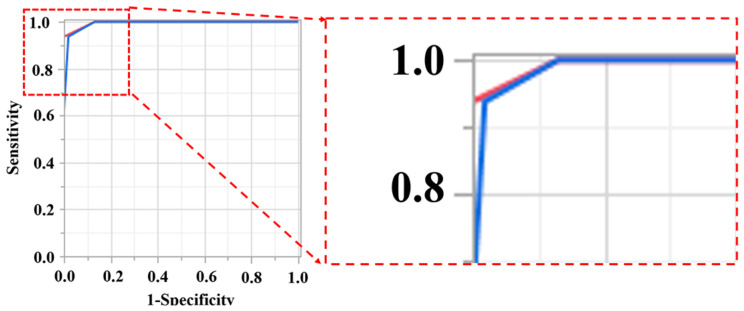
Compared results of ROC analysis between HDCTs reconstructed with hybrid-type IR and DLR methods (blue line: hybrid-type IR; red line: DLR). There was no significant difference in AUC between the two reconstruction methods (DLR: AUC = 0.996, hybrid-type IR: AUC = 0.993, *p* = 0.18).

Results of the ROC analysis are shown in Figure 5. Area under the curve (AUC) of DLR (AUC = 0.996) had no significant difference with hybrid-type IR (AUC = 0.993, *p* = 0.18), although the former was slightly larger than the latter. The compared diagnostic performance between two methods is shown in Table 4. There were no significant differences in sensitivity, specificity and accuracy between DLR and hybrid-type IR (*p* > 0.05).

## 4. Discussion

Our study results indicate that newly developed DLR, when compared with clinically applied hybrid-type IR on HDCT, could improve image quality and detailed evaluations of visibility of anatomical landmarks in middle and inner ears and temporal bone, although diagnostic performance was not affected. To the best of our knowledge, this study was the first to compare the capability of DLR for image quality and detailed evaluations of visibility of anatomical landmarks in middle and inner ears and temporal bone with that of hybrid-type IR on HDCT.

Comparisons of quantitative indexes on each of the CT data sets obtained with hybrid-type IR and DLR showed CT values and SNRs for the temporal bone, vestibule and cerebellar hemisphere for DLR were significantly higher than those for hybrid-type IR. These findings were compatible with those previously published by in vitro and in vivo studies [7,14,15]. The same studies reported that newly developed DLR improves image quality on not only conventional MDCT but also HDCT in various organs. Our results may therefore well be worth considering.

All κ values for each of the inter-observer agreements ranged between 0.61 and 1.0, and all inter-observer agreements were determined as ‘substantial’ or ‘almost perfect’. Our evaluations in this study can therefore be considered reproducible [23].

A comparison of image quality, artifacts and detailed evaluation of the visibility of anatomical landmarks in middle and inner ears and the temporal bone showed the use of DLR could result in significant improvement as compared with that of hybrid-type IR on HDCT. However, when comparing diagnostic performance between the above-mentioned two methods, there were no significant differences in AUC, sensitivity, specificity and accuracy between them. These results suggested that improved quantitative image quality gains with little clinical benefit for diagnosis were due to the following reasons: hybrid-type IR has already provided good image quality for clinical diagnosis, and DLR can reduce image noise more than hybrid-type IR and results in providing a sharper or clearer image as compared with DLR. Therefore, DLR may improve the confidence level for diagnosis level more than hybrid-type IR, although there was no significant difference in AUC between the two methods. Therefore, these facts suggest that DLR merits the use of HDCT data for improving image quality and visualization of anatomical landmarks in middle and inner ears and temporal bones, although the newly developed method had less influence on the diagnosis of otologic diseases in routine clinical practice. However, improved SNR on HDCT reconstructed with DLR may be possible to reduce radiation dose more than that with hybrid-type IR while keeping image quality and diagnostic performance. Therefore, DLR may impact radiation dose reduction as compared with hybrid-type IR, and further investigations are warranted to demonstrate the clinical relevance of DLR on low-dose CT in the near future.

There were several limitations to this study. First, the study cohort was limited, and the number of otologic disease patients was small. These limitations are likely to have affected our study results. Second, both reconstruction methods for HDCT were provided by a single vendor and not compared with those provided by other vendors. Moreover, we used only the HDCT system, not other CT systems such as ADCT and PCDCT, while other reconstruction methods such as MBIR and conventional DLR were not used either in this study. Therefore, this fact is affected by our study results, and the study results were not transferrable to other CT scanners provided not only by the same but also other vendors. In addition, the reconstruction matrix in this study was a 512 × 512 matrix and not assessed CT images with a 1024 × 1024 matrix and a 2048 × 2048 matrix because of larger data storage and limited clinical availability. Furthermore, a few investigators have suggested that PCDCT can be used for middle and inner ears as well as temporal bones. These considerations must thus also have affected our study results. Third, we did not directly compare its effect on decision-making for patients’ treatment. The clinical relevance of our findings for patients’ care has therefore not been determined, and further studies using a large prospective cohort with otologic diseases are warranted. Fourth, this study did not use an in vitro study, and detailed analyses for spatial resolution improvements were not analyzed in detail, while further investigations with phantoms are also warranted.

In conclusion, DLR showed superior potential to that of hybrid-type IR for better image quality and visualization of anatomical landmarks in middle and inner ears and temporal bones on HDCT, although diagnostic performance was not affected in clinical practice.

## Figures and Tables

**Table 1:** Patients’ characteristics.

Gender (Cases)	Male		69
	Female		71
Age (years)	All cases	Mean	61
		Range	11–86
	Male	Mean	62
		Range	18–84
	Female	Mean	60
		Range	11–86
Diagnosis (cases)	Without diseases		108
	With diseases		32
	Underlying diseases	Otitis media cholesteatoma	9
		Chronic otitis media	7
		Ossicular chain disruption	6
		Otosclerosis	5
		temporal bone fracture	5

**Table 2:** Inter-observer agreements for overall image quality and artifacts.

Qualitative Index	Reconstruction Method	Observers	Visual Score					κ	*p* Value
			1	2	3	4	5		
Overall image quality	DLR	1	0	0	0	22	194	0.61	<0.0001
		2	0	0	0	44	172		
	hybrid-type IR	1	0	0	0	98	118	0.63	<0.0001
		2	0	0	10	120	86		
Artifacts	DLR	1	194	22	0	0	0	1.0	<0.0001
		2	194	22	0	0	0		
	hybrid-type IR	1	118	98	0	0	0	0.63	<0.0001
		2	108	98	10	0	0		

DLR: deep learning reconstruction, IR: iterative reconstruction.

**Table 3:** Inter-observer agreements for detailed evaluation of the visibility of anatomical landmarks in middle and inner ears and temporal bone.

Anatomical Structure	Reconstruction Method	Observers	Visual Score					κ	*p* Value
			1	2	3	4	5		
Middle ear	DLR	1	0	0	0	33	183	0.80	<0.0001
		2	0	0	0	44	172		
	hybrid-type IR	1	0	0	10	120	86	0.62	<0.0001
		2	0	0	10	121	85		
Inner ear	DLR	1	0	0	0	22	194	0.62	<0.0001
		2	0	0	0	43	173		
	hybrid-type IR	1	0	0	11	87	118	0.73	<0.0001
		2	0	0	11	119	86		
Temporal bone	DLR	1	0	0	0	32	184	0.62	<0.0001
		2	0	0	0	33	183		
	hybrid-type IR	1	0	0	0	119	97	0.62	<0.0001
		2	0	0	10	121	85		

DLR: deep learning reconstruction, IR: iterative reconstruction.

**Table 4:** Compared diagnostic performance between two reconstruction methods.

Reconstruction Method	DLR	Hybrid-Type IR	*p* Value
Area under the curve (AUC)	0.996	0.993	0.18
Sensitivity (%)	93.8	93.8	1.00
	(30/32)	(30/32)	
Specificity (%)	100	98.1	0.50
	(108/108)	(106/108)	
Positive predictive value (%)	100	98.1	N/A
	(30/30)	(30/32)	
Negative predictive value (%)	98.2	98.1	N/A
	(108/110)	(106/108)	
Accuracy (%)	98.6	97.1	0.50
	(138/140)	(136/140)	

DLR: deep learning reconstruction, IR: iterative reconstruction, N/A: not applicable.

## Data Availability

The original contributions presented in this study are included in the article. Further inquiries can be directed to the corresponding author.
